# An explainable nomogram incorporating systemic inflammatory indicators and radiation esophagitis for risk assessment of radiation pneumonitis in lung cancer

**DOI:** 10.3389/fimmu.2026.1910553

**Published:** 2026-09-25

**Authors:** Jiayi Li, Chengqian Li, Jiamei Liu, Changkun Wang, Yingxue Zhao, Anqing Ge, Ying Dong, Xiangshan Yang

**Affiliations:** 1 School of Public Health, Jilin University, Changchun, China; 2 National Health Commission (NHC) Key Laboratory of Radiobiology, School of Public Health, Jilin University, Changchun, China; 3 Department of Radiation Oncology, Jilin Provincial Cancer Hospital, Changchun, China

**Keywords:** inflammatory indicators, lung cancer, nomogram, radiation pneumonitis, SHAP analysis

## Abstract

**Purpose:**

This study aimed to evaluate the value of systemic inflammatory prognostic markers and early clinical toxicity in the risk assessment of radiation pneumonitis (RP) after radiotherapy in lung cancer patients. We developed and validated an explainable, auxiliary nomogram integrating multiple features and SHAP analysis to optimize early risk monitoring and clinical decision-making.

**Methods:**

A least absolute shrinkage and selection operator (LASSO) penalized Cox model was used for feature selection to build a multivariable Cox proportional hazards model, which was visualized as a nomogram. A Fine-Gray competing risks model was used for sensitivity analysis. Model performance was evaluated using the C-index, 6-month time-dependent AUC, calibration, continuous NRI/IDI, and decision curve analysis (DCA). Internal validation using bootstrap resampling. Finally, SHAP analysis was performed to interpret the final Cox model through beeswarm and waterfall plots.

**Results:**

This study retrospectively collected data on 726 patients with lung cancer who received radiotherapy. The primary endpoint was grade ≥2 RP, with non-RP-related death treated as a competing risk. Multivariable Cox regression, validated by Fine-Gray competing risk analysis, identified alcohol history, radiotherapy modality, baseline lactate dehydrogenase (LDH), systemic inflammation score (SIS), and acute radiation esophagitis (RE) as independent factors (all P < 0.05). Among the three sequential models, the integrated model (Model 3) achieved the highest C-index (0.688) and 6-month AUC (0.713). Compared to the baseline model (Model 1), Model 3 improved risk reclassification (continuous NRI = 0.274, IDI = 0.037; both P < 0.05). Model 3 demonstrated optimal calibration and yielded the highest net clinical benefit within the 10%-15% threshold probability range in DCA. Furthermore, SHAP analysis enhanced model interpretability, indicating a potential association between a baseline systemic inflammation score of zero (SIS = 0) and higher RP risk.

**Conclusion:**

This study developed and validated a nomogram that integrates fundamental clinical factors with inflammatory indicators (LDH and SIS) and early acute radiation esophagitis (RE). This model serves as a multidimensional, auxiliary screening tool for early risk assessment of radiation pneumonitis (RP) in lung cancer patients.

## 1 Introduction

Thoracic malignancies, especially lung cancer, are among the malignant tumors with the highest incidence and mortality rates worldwide (1). Radiotherapy (RT) is the standard treatment modality for non-small cell lung cancer (NSCLC) and limited-stage small cell lung cancer (SCLC) (2). Radiation pneumonitis (RP) is one of the most potentially life-threatening, dose-limiting toxicities after thoracic radiotherapy for lung cancer. It is especially common during radical radiotherapy or postoperative adjuvant radiotherapy for lung cancer patients (3). As a common acute complication after thoracic radiotherapy, it can lead to severe lung function impairment. In severe cases, it can develop into irreversible pulmonary fibrosis, which negatively affects the prognosis and quality of life of patients (4, 5). In recent years, the clinical integration of multi-modality therapies, including chemotherapy, targeted agents, and immunotherapy, has substantially improved survival outcomes for patients with lung cancer (6, 7). However, under these comprehensive treatment paradigms, the incidence of treatment-related pulmonary toxicities, particularly radiation pneumonitis, has also risen, presenting a substantial clinical challenge (8, 9).

With the accumulation of real-world data, it is necessary to establish predictive systems that complement existing standards. Therefore, the aim of this study was to develop and validate a predictive model based solely on routine clinical and hematological indices. Systemic inflammatory and immune-related biomarkers reflect individual biological responses to radiation and may contribute additional information for RP risk assessment. A growing body of evidence indicates that the pathogenesis of RP is fundamentally a systemic immune inflammatory cascade reaction triggered by ionizing radiation (10–12). This process involves multiple complex mechanisms, including DNA damage and apoptosis, lymphocyte exhaustion, neutrophil and monocyte infiltration, and sustained release of pro-inflammatory and profibrotic cytokines (13–16). These characteristics suggest that systemic inflammatory biomarkers can provide valuable prognostic information.

In recent years, systemic inflammatory composite indices derived from peripheral blood white blood cell counts, including the neutrophil-to-lymphocyte ratio (NLR), derived NLR (dNLR), platelet-to-lymphocyte ratio (PLR), systemic immune inflammatory index (SII), systemic inflammatory response index (SIRI), and systemic inflammation score (SIS), have demonstrated significant prognostic value for predicting outcomes of various malignancies, owing to their easy accessibility and repeatability (17–20). For example, in a machine learning-based analysis of non-small cell lung cancer (NSCLC), Wang et al. found that incorporating the neutrophil-to-lymphocyte ratio at week 4 of radiotherapy (NLR-4w) after integrating dosimetric, clinical, and radiomic features led to a small but statistically significant increase in the model’s AUC value, rising from 0.918 to 0.938 (21). In addition, Qiu et al. found that SIRI is an independent factors of grade ≥2 radiation esophagitis in patients with small cell lung cancer (SCLC) undergoing chemoradiotherapy (22).

To this end, this study developed an interpretable nomogram for RP risk assessment in a real-world cohort of lung cancer patients undergoing radiotherapy. Candidate systemic inflammatory indicators were screened using LASSO, followed by sequential modeling to evaluate their incremental value. Model performance was assessed within a competing-risk framework, and SHAP analysis was used to enhance model interpretability.

## 2 Materials and methods

### 2.1 Patients

This retrospective, single-center cohort study was approved by the Institutional Review Board for Science and Technology Ethics of Jilin Provincial Cancer Hospital (approval number: 202511-011-01), and the requirement for informed consent was waived. We recruited 726 lung cancer patients who received thoracic radiotherapy at Jilin Provincial Cancer Hospital between January 2015 and December 2025.

The inclusion criteria were as follows: (A) Cytologically or histopathologically confirmed lung cancer (including both NSCLC and SCLC); (B) Receiving initial thoracic radiotherapy; (C) An Eastern Cooperative Oncology Group performance status (ECOG PS) score of 0 to 2; (D) Follow-up time ≥ 6 months; (E) Having complete baseline data, as well as complete hematological and biochemical examination data. The exclusion criteria were: (A) A previous history of thoracic radiotherapy (including radiotherapy for breast or lung mediastinal tumors); (B) Active lung inflammation or autoimmune diseases before radiotherapy; (C) Severe underlying heart disease; (D) Concurrent presence of other malignant tumors. The workflow of data filtration is displayed in **Figure 1**.

**Figure 1:**
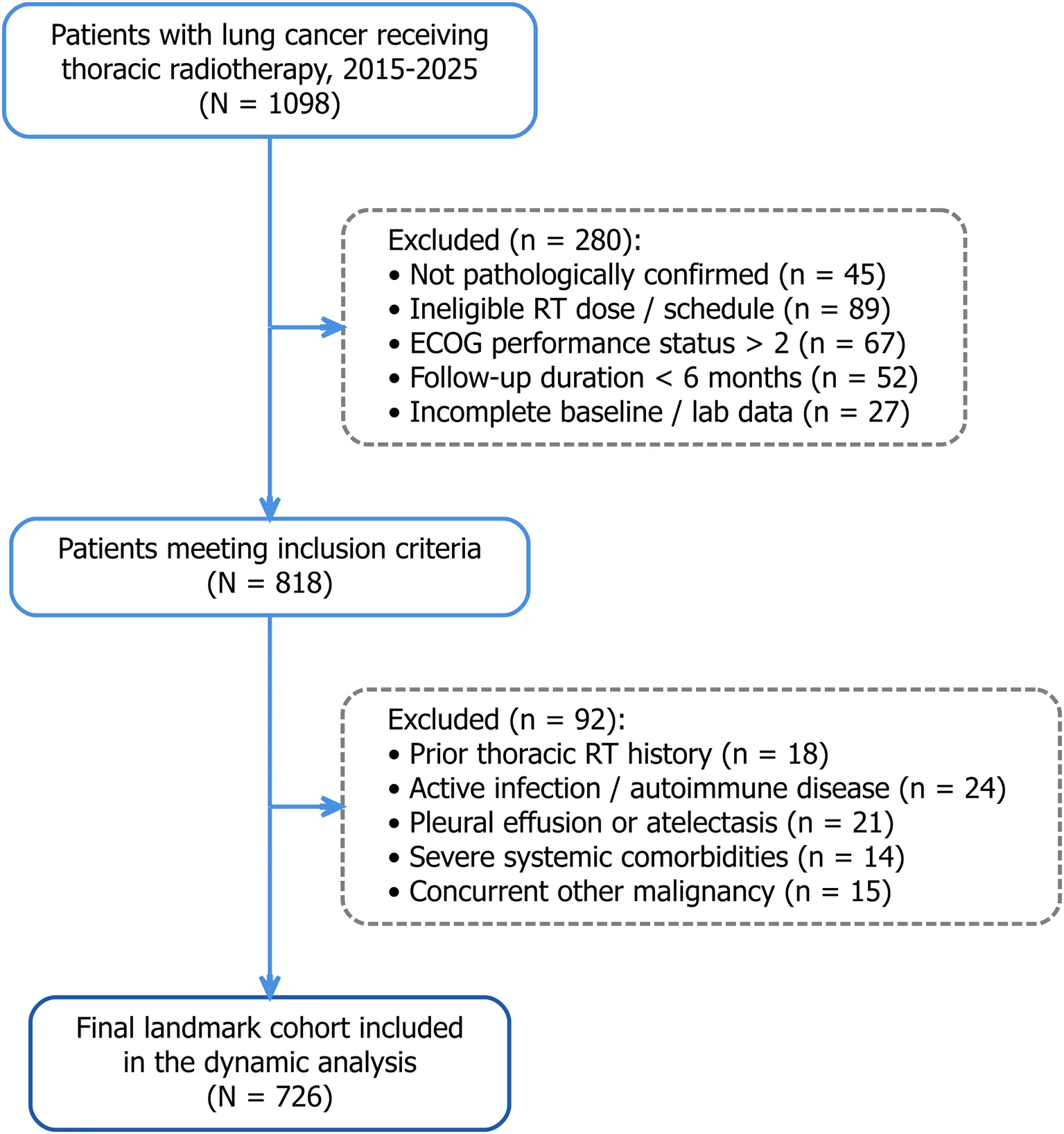
The workflow of the data filtration.

### 2.2 Definition of endpoint events

Referring to the Common Terminology Criteria for Adverse Events (CTCAE 5.0) of the National Cancer Institute (NCI), the following conditions can be diagnosed as grade ≥2 RP: (A) Clarify the history of thoracic radiotherapy. RP usually occurs 1–6 months after the start of radiotherapy; (B) Chest CT performance, limited to patchy shadows, stripy shadows, lung consolidation, or honeycomb-like changes in the irradiated area of the lungs, which may be accompanied by air bronchograms, and a few patients have imaging changes outside the irradiated area; (C) Clinical symptoms, new or aggravated cough, sputum, chest tightness, shortness of breath, fever ≥ 38°C following radiotherapy; (D) Treatment requirements, need to receive oral or intravenous glucocorticoids (23). The diagnosis was independently determined by two radiotherapists with more than 5 years of clinical experience. At the time of disagreement, they were arbitrated by senior physicians (associate chief physicians or above) in the respiratory department, who recorded the time of RP occurrence from the first day of radiotherapy to the date of diagnosis.

To account for the time-dependent nature of acute radiation esophagitis (RE), a prespecified landmark analysis was performed with the landmark time set at 30 days after the initiation of radiotherapy. RE was graded according to CTCAE version 5.0 at the landmark time and entered into the Cox proportional hazards model as a fixed covariate. We verified the absence of RP or death before the landmark time by examining the recorded dates of RP onset and death for all eligible patients, confirming that no patient experienced either event before day 30, thereby ensuring that RE status was determined prior to outcome occurrence.

### 2.3 Feature selection and sequential model construction

A two-stage hybrid strategy was employed to select the final indicators for the multivariable model. First, a least absolute shrinkage and selection operator (LASSO) penalized Cox regression with an alpha parameter of 1.0 was performed on all candidate variables. This data-driven approach identified ten candidate factors: alcohol history, esophagitis, radiotherapy modality, clinical subtype, T stage, metastatic status, hemoglobin, albumin, lactate dehydrogenase, and systemic inflammation score. In the second stage, this list was refined based on clinical and statistical expertise to minimize multicollinearity and maintain model parsimony. Specifically, albumin was excluded because its values are mathematically nested within the calculation of the Systemic Inflammation Score. Similarly, metastatic status and T stage were omitted due to their clinical and conceptual collinearity with the overall clinical subtype variable. Although hemoglobin was initially retained for multivariable testing, it demonstrated no statistically significant independent prognostic value in the multivariable analysis, with a hazard ratio of 1.01 (95% confidence interval: 0.99 to 1.02, P = 0.383). Consequently, to maintain model parsimony and focus on the systemic inflammatory and tissue damage markers, hemoglobin was subsequently excluded from the final model. Subsequently, total radiation dose was re-included as a clinically crucial *a priori* variable. Finally, the remaining subset of clinical, biological, and toxicological indicators was integrated to construct the final multivariable Cox proportional hazards model.

The Systemic Inflammation Score was calculated based on baseline serum albumin and the lymphocyte-to-monocyte ratio, defined as the absolute lymphocyte count divided by the absolute monocyte count. Patients were assigned a score of 0 for albumin of 40 g/L or higher and a ratio of 2.0 or higher, 2 when both values fell below these thresholds, and 1 for either abnormality.

To evaluate the incremental predictive value of these distinct feature sets, three sequential progressive models were constructed based on the standard Cox proportional hazards framework: Model 1 (Base Clinical): Incorporates fundamental clinical and treatment characteristics (total radiation dose, clinical subtype, alcohol history, and radiotherapy modality). Model 2 (+ Biomarkers): Builds upon Model 1 by integrating baseline systemic inflammatory indicators (LDH per 50 U/L increment^1^ and SIS). Model 3 (+ Early Toxicity): Represents the ultimate comprehensive model, building upon Model 2 by further incorporating the occurrence of early acute toxicity (radiation esophagitis).

### 2.4 Statistical analysis

Missing values for clinical and hematological variables were handled using multiple imputation by chained equations (MICE). Continuous variables were presented as the median (interquartile range, IQR) and compared using the Wilcoxon rank-sum test. Categorical variables were presented as frequencies and compared using the Pearson chi-square test or Fisher’s exact test. A sensitivity analysis was performed by refitting Model 3 using the Fine-Gray subdistribution hazards model. Cumulative incidence function (CIF) curves were plotted to visualize event probabilities. Model performance was comprehensively evaluated using multiple metrics. Discrimination was assessed by the 6-month time-dependent area under the ROC curve (AUC). For internal validation, we performed 1,000 bootstrap resamples to estimate Harrell’s C-index, generate calibration curves, and calculate the Brier score. The incremental predictive value of the model was further quantified using the continuous net reclassification improvement (NRI) and integrated discrimination improvement (IDI). Clinical utility was evaluated with decision curve analysis (DCA). Finally, the SHAP analysis was employed to generate beeswarm and waterfall plots. All statistical analyses were performed using R software (version 4.5.3). A two-sided P < 0.05 was considered statistically significant.

## 3 Result

### 3.1 Baseline characteristics

This study included 726 lung cancer patients who received thoracic radiotherapy. During the follow-up period, a total of 86 patients had grade ≥2 RP, with an overall incidence of 11.8%. The median time to the onset of RP for the entire cohort was 121.5 days. The median age of the cohort was 66 years old. The patients were mainly male (68.0%) and presented with advanced-stage diseases (stage III-IV accounted for 87.6%; stage N2-N3 accounted for 77.0%). Small cell carcinoma (51.1%) and squamous cell carcinoma (33.2%) were the most common pathological types. In terms of treatment regimen, most patients received chemotherapy (71.9%), and volumetric modulated arc therapy (VMAT, 71.9%), and the median total radiotherapy dose was 60 Gy.

The proportion of patients in the RP group with a history of alcohol was significantly higher (65.1% vs. 53.1%, P = 0.038), and the incidence of acute radiation esophagitis (RE) was approximately twice as high in the RP group (24.4% vs. 13.4%, P = 0.014). There were no significant differences between the two groups in gender, BMI, underlying lung diseases, tumor location, pathological type, or main systemic treatment methods (chemotherapy, immunotherapy, targeted treatment; all P > 0.05). **Table 1** for details of the baseline characteristics of patients. For continuous inflammatory biomarkers, optimal cutoff values were determined using the Youden Index derived from receiver operating characteristic (ROC) curve analysis, and the resulting patient distribution is presented in **Supplementary Table S1**^^2^^.

**Table 1:** Baseline characteristics of all patients.

Characteristic	Total (N = 726)^1^	With RP N = 86^1^	Without RP N = 640^1^	*P* value^2^
Age	66 (60-71)	63 (57-70)	66 (60-71)	0.050
Gender				0.713
Male	494 (68.0%)	57 (66.3%)	437 (68.3%)	
Female	232 (32.0%)	29 (33.7%)	203 (31.7%)	
BMI (kg/m²)				0.577
< 18.5	36 (5.0%)	6 (7.0%)	30 (4.7%)	
18.5-24	327 (45.0%)	35 (40.7%)	292 (46%)	
24-28	263 (36.2%)	31 (36.0%)	232 (36%)	
≥ 28	100 (13.8%)	14 (16.3%)	86 (13%)	
Alcohol History				0.038
Yes	396 (54.5%)	56 (65.1%)	340 (53.1%)	
No	330 (45.5%)	30 (34.9%)	300 (46.9%)	
Obstructive Pneumonia				>0.999
Yes	222 (30.6%)	26 (30.2%)	196 (30.6%)	
No	504 (69.4%)	60 (69.8%)	444 (69.4%)	
Surgery				0.796
Yes	38 (5.2%)	5 (5.8%)	33 (5.2%)	
No	688 (94.8%)	81 (94.2%)	607 (94.8%)	
Chemotherapy				0.523
Yes	522 (71.9%)	59 (68.6%)	463 (72.3%)	
No	204 (28.1%)	27 (31.4%)	177 (27.7%)	
Immunotherapy				0.396
Yes	241 (33.2%)	32 (37.2%)	209 (32.7%)	
No	485 (66.8%)	54 (62.8%)	431 (67.3%)	
Targeted Therapy				0.364
Yes	194 (26.7%)	19 (22.1%)	175 (27.3%)	
No	532 (73.3%)	67 (77.9%)	465 (72.7%)	
Endocrine Therapy				0.079
Yes	509 (70.1%)	53 (61.6%)	456 (71.3%)	
No	217 (29.9%)	33 (38.4%)	184 (28.8%)	
Esophagitis				0.014
Yes	107 (14.7%)	21 (24.4%)	86 (13.4%)	
No	619 (85.3%)	65 (75.6%)	554 (86.6%)	
RT Modality				0.014
IMRT	82 (11.3%)	7 (8.1%)	75 (11.7%)	
VMAT	522 (71.9%)	55 (64.0%)	469 (73.3%)	
TOMO	122 (16.8%)	24 (27.9%)	96 (15.0%)	
Total Dose (Gy)	60 (50-60)	54 (50-60)	60 (50-60)	0.104
Tumor Location				0.808
Upper lobe	401 (55.2%)	48 (55.8%)	353 (55.2%)	
Middle lobe	53 (7.3%)	8 (9.3%)	45 (7.0%)	
Lower lobe	207 (28.5%)	24 (27.9%)	183 (28.6%)	
Other^3^	65 (9.0%)	6 (7.0%)	59 (9.2%)	
Pathology Type				0.652
Adenocarcinoma	100 (13.8%)	11 (12.8%)	89 (13.9%)	
Squamous cell carcinoma	241 (33.2%)	34 (39.5%)	207 (32.3%)	
Small cell carcinoma	371 (51.1%)	40 (46.5%)	331 (51.7%)	
Other	14 (1.9%)	1 (1.2%)	13 (2.0%)	
Clinical Subtype				0.197
I-II	90 (12.4%)	9 (10.5%)	81 (12.7%)	
III	535 (73.7%)	70 (81.4%)	465 (72.7%)	
IV	101 (13.9%)	7 (8.1%)	94 (14.7%)	
T Stage				0.487
1	83 (11.4%)	7 (8.1%)	76 (11.9%)	
2	260 (35.8%)	37 (43.0%)	223 (34.8%)	
3	153 (21.1%)	17 (19.8%)	136 (21.3%)	
4	230 (31.7%)	25 (29.1%)	205 (32.0%)	
N Stage				0.968
0	94 (12.9%)	10 (11.6%)	84 (13.1%)	
1	73 (10.1%)	9 (10.5%)	64 (10.0%)	
2	368 (50.7%)	43 (50.0%)	325 (50.8%)	
3	191 (26.3%)	24 (27.9%)	167 (26.1%)	
Metastatic				0.133
Yes	101 (13.9%)	7 (8.1%)	94 (14.7%)	
No	625 (86.1%)	79 (91.9%)	546 (85.3%)	
WBC	5.5 (4.0, 7.4)	5.5 (4.1, 6.5)	5.5 (4.0, 7.5)	0.554
Hb	123.6 (112.3, 134.4)	128.0 (114.6, 135.9)	123.3 (112.3, 134.0)	0.122
ALC	1.6 (1.2, 1.9)	1.6 (1.2, 1.9)	1.6 (1.2, 2.0)	0.949
ANC	3.2 (2.1, 4.8)	3.0 (2.2, 4.4)	3.2 (2.1, 4.9)	0.480
PLT	210.5 (163.0, 265.0)	206.5 (167.0, 270.0)	211.0 (162.0, 265.0)	0.978
MONO	0.5 (0.4, 0.7)	0.5 (0.4, 0.7)	0.5 (0.4, 0.7)	0.999
BASO	0.0 (0.0, 0.0)	0.0 (0.0, 0.0)	0.0 (0.0, 0.0)	0.759
EOS	0.1 (0.0, 0.1)	0.1 (0.0, 0.1)	0.1 (0.0, 0.1)	0.884
GLU	5.5 (5.0, 6.2)	5.4 (5.1, 6.2)	5.5 (5.0, 6.2)	0.766
ALB	42.2 (39.7, 44.3)	43.2 (41.4, 45.3)	42.1 (39.6, 44.2)	0.002
LDH	201.2 (177.0, 232.4)	207.7 (184.0, 241.0)	199.0 (175.8, 230.1)	0.023
CRE	69.7 (59.9, 80.5)	67.1 (60.7, 77.8)	70.0 (59.8, 80.8)	0.410
INR	1.0 (0.9, 1.0)	1.0 (0.9, 1.0)	1.0 (0.9, 1.0)	0.916
NLR	2.0 (1.3, 3.3)	2.0 (1.3, 3.5)	2.0 (1.3, 3.3)	0.708
dNLR	1.5 (1.0, 2.2)	1.5 (1.0, 2.2)	1.5 (1.0, 2.2)	0.623
SIRI	1.0 (0.6, 1.8)	1.0 (0.6, 1.6)	1.0 (0.6, 1.8)	0.784
MLR	0.3 (0.2, 0.4)	0.3 (0.2, 0.4)	0.3 (0.2, 0.4)	0.863
dMLR	0.1 (0.1, 0.1)	0.1 (0.1, 0.1)	0.1 (0.1, 0.1)	0.731
PLR	136.7 (101.8, 187.6)	135.8 (103.7, 196.9)	136.8 (101.3, 185.2)	0.905
SII	427.1 (260.4, 751.7)	431.8 (275.7, 735.6)	427.1 (252.9, 752.6)	0.916
PNI	49.8 (46.9, 53.1)	50.7 (48.6, 53.5)	49.7 (46.7, 53.1)	0.045
PAR	5.0 (3.9, 6.4)	5.0 (3.7, 6.2)	5.0 (3.9, 6.4)	0.639
ALI	49.5 (29.8, 76.7)	50.9 (33.8, 79.3)	49.4 (29.4, 75.8)	0.408
PIV	205.9 (106.3, 404.4)	202.4 (132.9, 353.8)	206.2 (105.2, 411.1)	0.988
LIPI				0.450
Good	542 (75%)	62 (72%)	480 (75%)	
Intermediate	153 (21%)	22 (26%)	131 (20%)	
Poor	31 (4.3%)	2 (2.3%)	29 (4.5%)	
SIS				0.019
Score 0	449 (62%)	65 (76%)	384 (60%)	
Score 1	226 (31%)	18 (21%)	208 (33%)	
Score 2	51 (7.0%)	3 (3.5%)	48 (7.5%)	

1 Median (Q1-Q3); n (%);.

2 Wilcoxon rank sum test; Fisher’s exact test;.

3 This category represents central tumors in the main bronchus, tumors originating in mediastinal locations, or tumors involving overlapping multi-lobar regions that could not be categorized into a single specific lobe.

### 3.2 Multivariable Cox regression and fine-gray sensitivity analysis

To prevent overfitting and eliminate multicollinearity among high-dimensional baseline features, a 10-fold cross-validated LASSO penalized Cox regression (α=1) was performed to screen for candidate factors (**Figure 2**).

**Figure 2:**
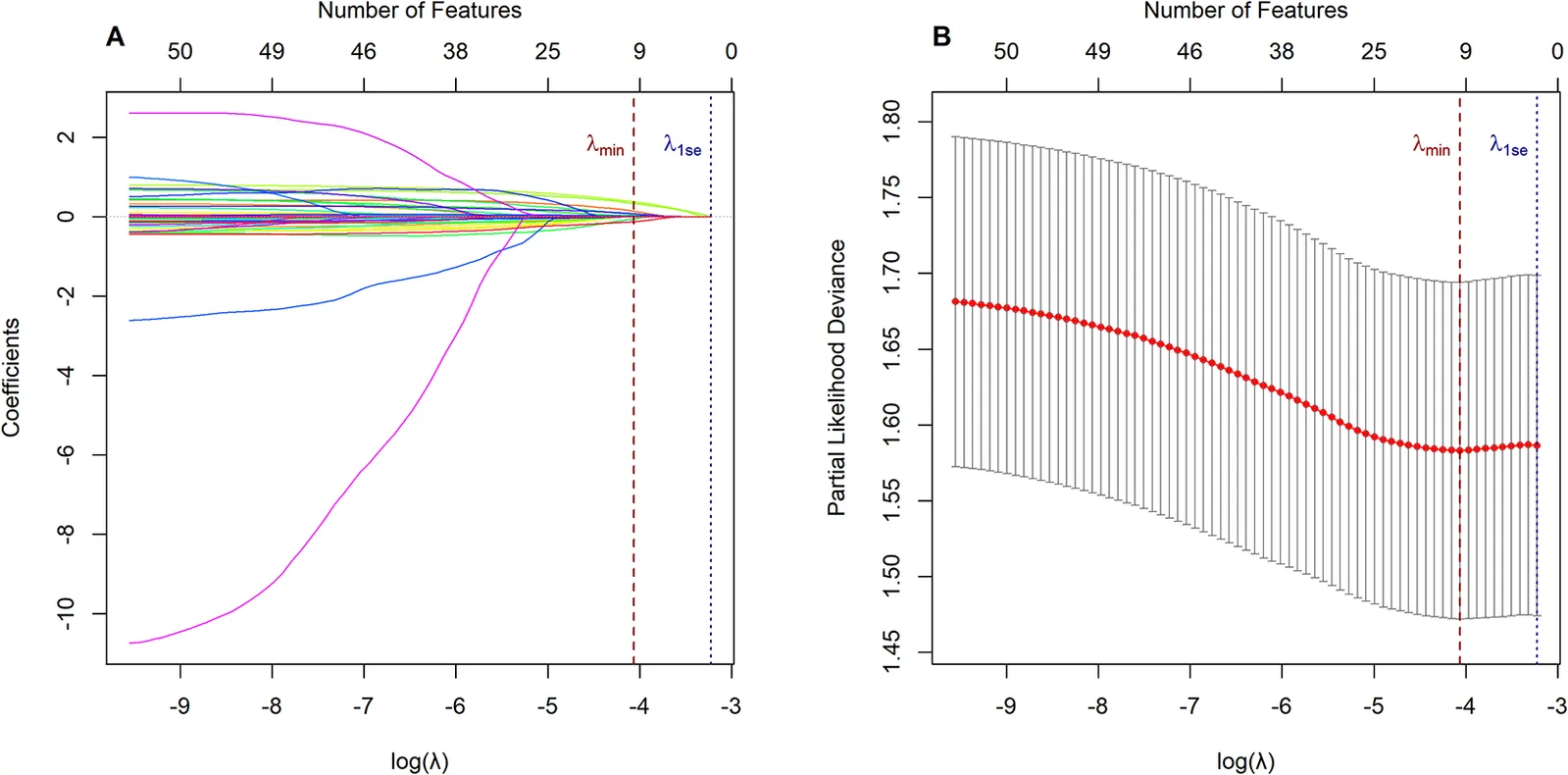
Feature selection using LASSO penalized Cox regression model (α = 1). **(A)** Coefficient profiles of candidate factors plotted against log(λ). The vertical dark red and dark blue dashed lines indicate the optimal λ_min_ and λ_1se_, respectively. **(B)** Tuning parameter selection using ten-fold cross-validation, plotting partial likelihood deviance against log(λ). Red dots show average deviance, and vertical bars represent standard errors. The dashed lines mark the optimal λ_min_ of 0.0172, which selected 10 features, and the λ_1se_ of 0.0397. To balance predictive performance and model parsimony, λ_min_ was chosen as the optimal threshold.

The main multivariate analysis of feature selection and nomogram construction was performed using the standard Cox proportional hazards model to estimate hazard ratios (HRs). As detailed in the primary Cox proportional hazards regression (**Table 2**, left panel), alcohol history (HR = 1.63, P = 0.039), TOMO radiotherapy modality (vs. VMAT: HR = 2.16, P = 0.002), elevated baseline LDH levels (HR = 1.24 per 50 U/L increment, P = 0.007), an optimal Systemic Inflammation Score (SIS = 0; HR = 0.59, P = 0.013), and the occurrence of acute radiation esophagitis (HR = 1.85, P = 0.017) emerged as independent risk factors. Detailed results of the univariable Cox proportional hazards regression are provided in **Supplementary Table S2**.

**Table 2:** Multivariable Cox and fine-gray competing risk analyses for the radiation pneumonitis.

Variable	Cox proportional hazards		Fine-gray competing risk	
	HR (95% CI)	*P* value	sHR (95% CI)	*P* value
Total Dose (Gy)	0.99 (0.94, 1.03)	0.604	0.99 (0.95, 1.03)	0.594
Clinical subtype				
I-II	Reference		Reference	
III	1.13 (0.56, 2.28)	0.734	1.14 (0.58, 2.27)	0.702
IV	0.56 (0.20, 1.52)	0.253	0.56 (0.21, 1.54)	0.265
Alcohol History	1.63 (1.02, 2.59)	0.039	1.63 (1.05, 2.55)	0.031
Radiotherapy modality				
VAMT	Reference		Reference	
IMRT	0.85 (0.38, 1.90)	0.699	0.85 (0.39, 1.86)	0.693
TOMO	2.16 (1.33, 3.51)	0.002	2.17 (1.35, 3.48)	0.001
LDH^1^	1.24 (1.06, 1.45)	0.007	1.24 (1.08, 1.42)	0.002
SIS	0.59 (0.38, 0.89)	0.013	0.59 (0.39, 0.89)	0.012
Esophagitis	1.85 (1.11, 3.06)	0.017	1.83 (1.10, 3.06)	0.020

1 LDH was analyzed as a continuous variable divided by 50 to facilitate clinical interpretation; thus, the corresponding HR and sHR represent the relative risk increase per 50 U/L increment in baseline LDH levels.

CI, confidence interval; HR, hazard ratio (from standard Cox proportional hazards regression); sHR, subdistribution hazard ratio (from Fine-Gray competing risk model).

Sensitivity analysis was conducted using the Fine-Gray Subdistribution Risk Model (**Table 2**, right panel; and **Supplementary Table S3**). Cumulative Incidence Function (CIF) curves, evaluated by Gray’s test, were used to visualize the incidence of RP across different subgroups (**Figure 3**). Receiver operating characteristic (ROC) curves of candidate variables in **Supplementary Figure S1**. The CIF curves for other univariate indicators are provided in the **Supplementary Figure S2**.

**Figure 3:**
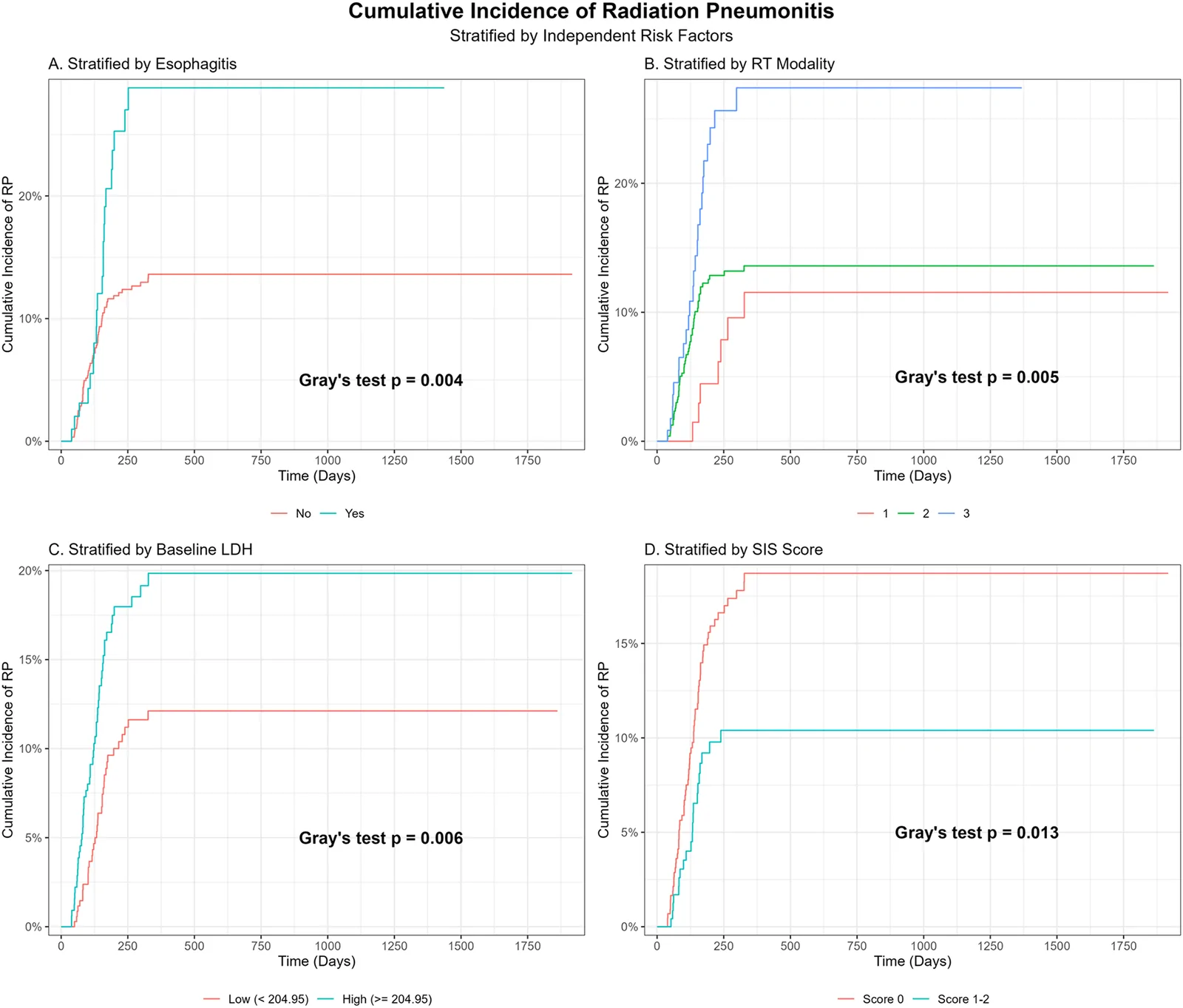
Cumulative incidence functions (CIF) of radiation pneumonitis stratified by independent risk factors. The curves illustrate the cumulative probability of developing grade ≥2 radiation pneumonitis (RP) over time, accounting for the competing risk of death. Patients are stratified according to the core prognostic parameters identified in the multivariable analysis: **(A)** acute esophagitis (No vs. Yes); **(B)** radiotherapy modality (IMRT vs. VAMT vs. TOMO); **(C)** baseline lactate dehydrogenase (LDH) level (Low < 204.95 U/L vs. High ≥ 204.95 U/L, stratified by the optimal cut-off value determined via receiver operating characteristic curve analysis); and **(D)** Systemic Inflammation Score (SIS, Score 0 vs. Score 1-2).

Since patients may die from other causes before RP occurs, death without RP was considered a competing risk. In this cohort, the incidence of such competing events was extremely low (3.9%). When the incidence of competing events is low, Cox models generally yield estimates comparable to those of competing risk analysis and remain appropriate because of their interpretability (24). Nevertheless, in order to ensure absolute statistical rigor, we still conduct the Fine-Gray subdistribution hazard model for sensitivity analysis to calculate sHRs (25). A parallel sensitivity analysis using the Fine-Gray subdistribution hazards model was executed (**Table 2**, right panel). The Fine-Gray analysis yielded concordant results, with the subdistribution hazard ratios (sHRs) closely matching the standard HRs across all key variables. The consistency between the Cox and Fine-Gray models confirms the robustness of the indicators we have identified.

### 3.3 Comparison of nomogram construction and incremental model performance

Based on the standard Cox proportional hazards model, the core variables were incorporated into a multivariable regression to construct a visual nomogram (**Figure 4**), enabling clinicians to rapidly evaluate individualized RP risk. To quantify the true contribution of baseline systemic inflammatory indicators and early clinical toxicity in the risk of radiation pneumonitis (RP), we developed three sequential models and conducted a multidimensional performance evaluation.

**Figure 4:**
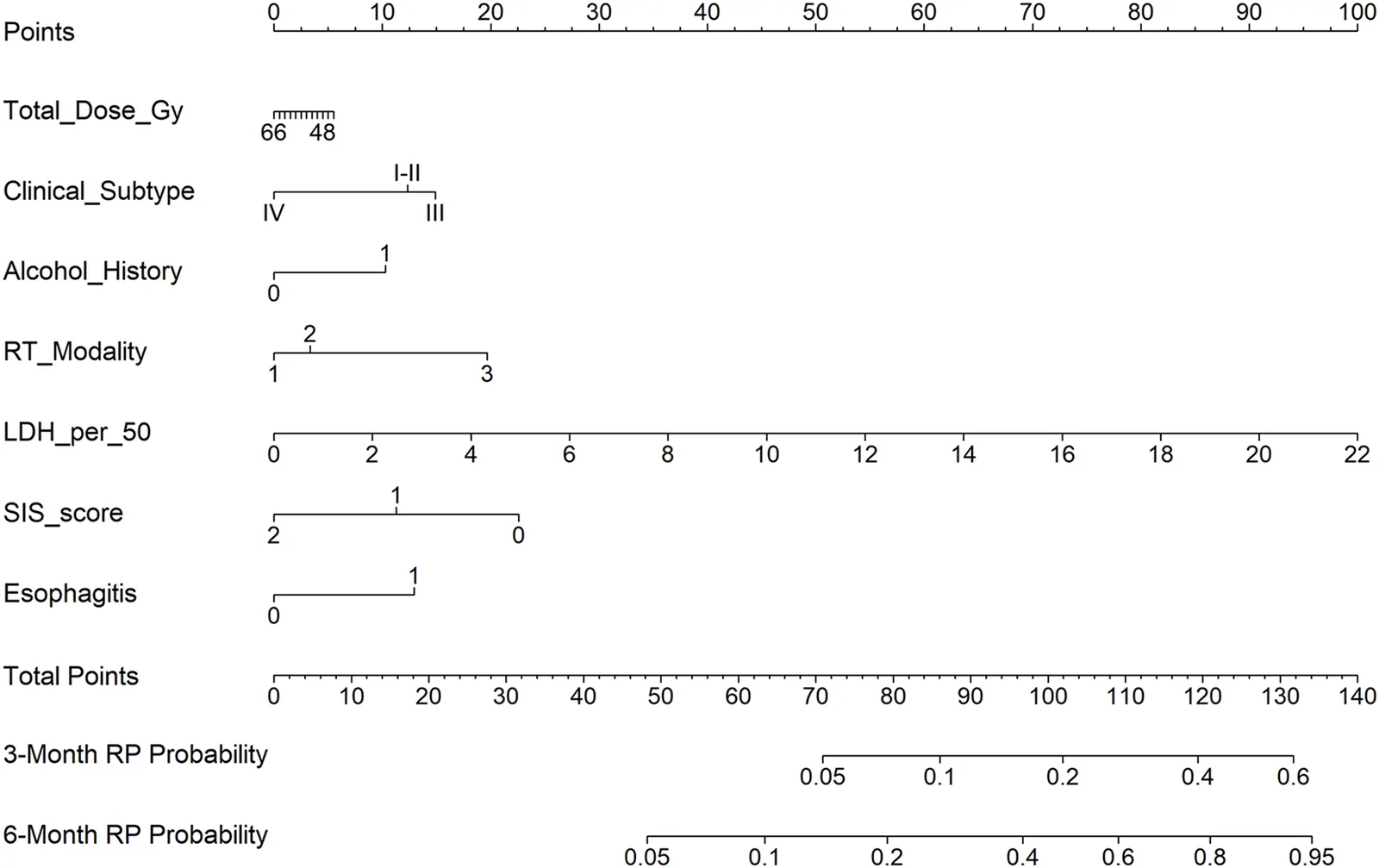
Nomogram for predicting the 3-month and 6-month probabilities of severe radiation pneumonitis (RP). The predictive nomogram was constructed based on the comprehensive multivariable Cox proportional hazards model (Model 3). It integrates seven independent prognostic factors: total radiation dose (Gy), clinical subtype (I-II, III, IV), alcohol history (0 = No, 1 = Yes), radiotherapy modality (1 = IMRT, 2 = VMAT, 3 = TOMO), baseline LDH level (per 50 U/L increment), systemic inflammation score (SIS, Score 0 vs. Score 1-2), and the occurrence of acute esophagitis (0 = No, 1 = Yes).

In order to evaluate the incremental discriminative capacity conferred by the newly identified systemic biomarkers, time-dependent ROC curves were generated at the 6-month clinical milestone (**Figure 5**). A comprehensive comparison of the three progressive models is detailed in **Table 3**. Furthermore, this step-wise enhancement across all evaluated metrics is visually presented in a multidimensional radar plot (**Figure 6**).

**Figure 5:**
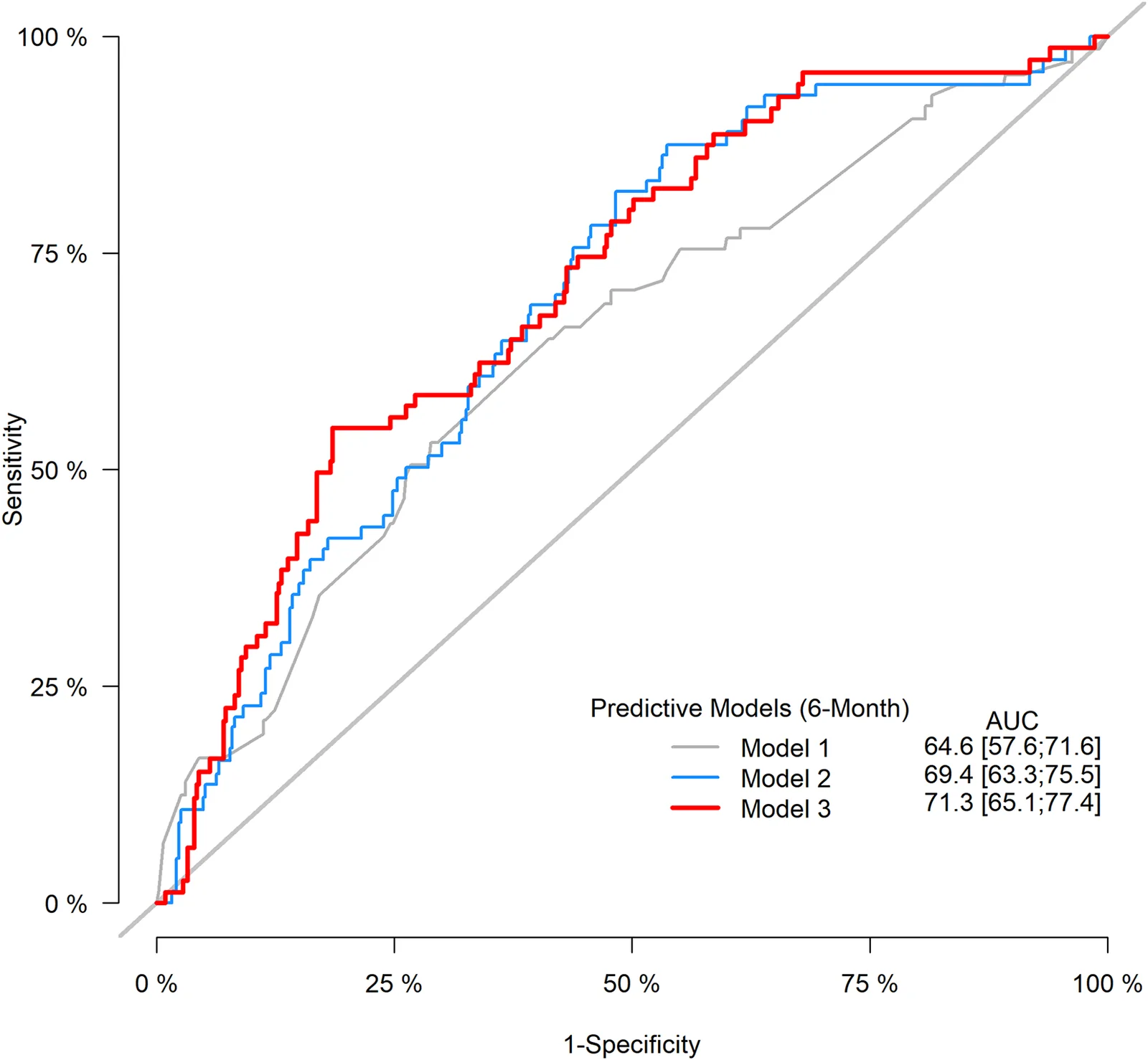
Comparison of time-dependent receiver operating characteristic (ROC) curves among the three sequential predictive models at 6 months. The grey line represents Model 1, the blue line denotes Model 2, and the red line depicts the comprehensive Model 3. The respective Area Under the Curve (AUC) values and 95% Confidence Intervals (CI) are detailed in the lower right corner. The substantial upward shift of the red curve visually corroborates the significant incremental discriminative value of the newly incorporated biomarkers.

**Figure 6:**
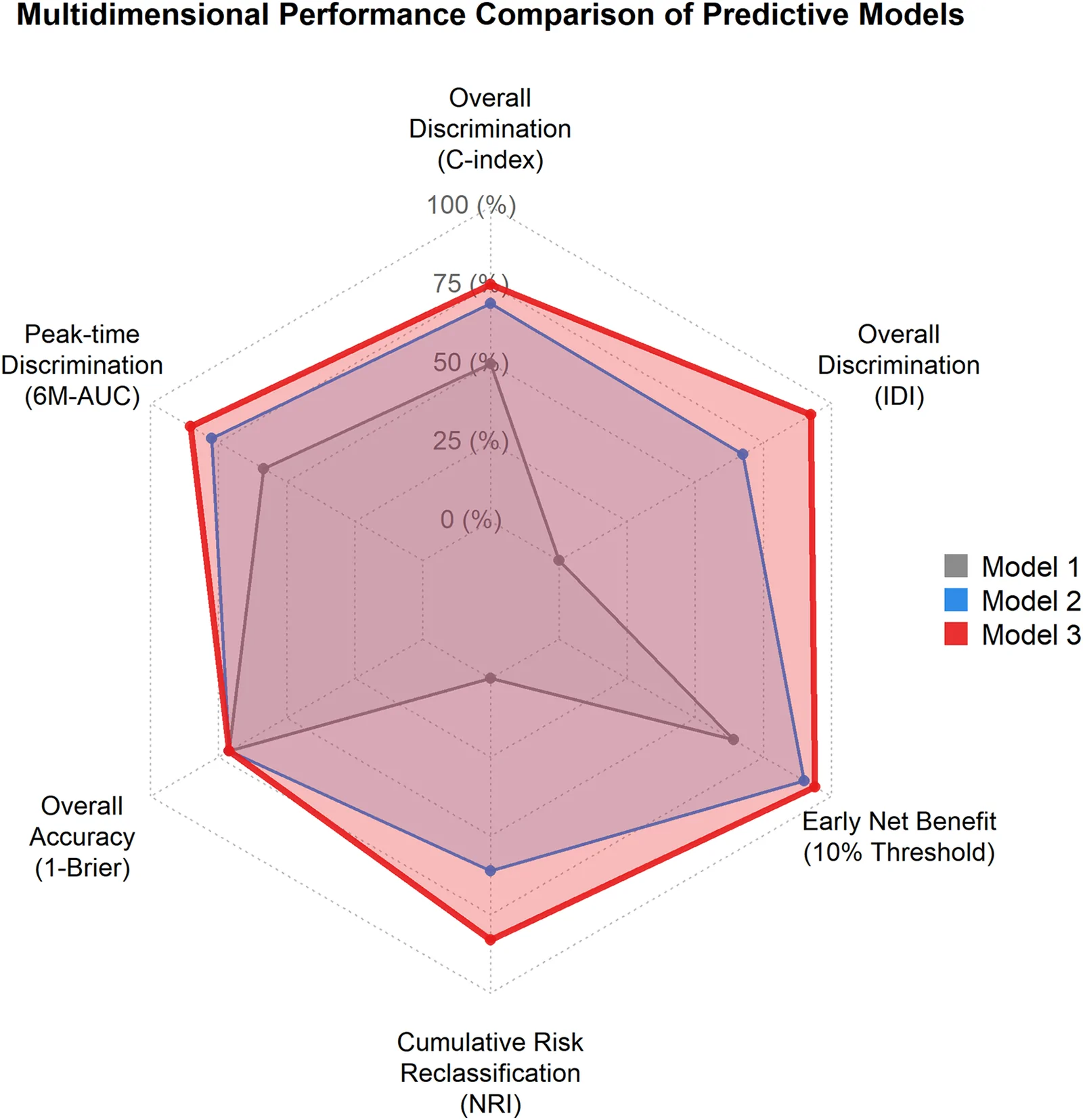
Multidimensional performance radar plot of the sequential predictive models. The performance of the model is compared across six metrics, scaled from center (minimum) to perimeter (maximum): (1) Overall Discrimination (C-index); (2) Peak-time Discrimination (6-month AUC); (3) Overall Accuracy (1 - Brier score); (4) Cumulative Risk Reclassification (continuous NRI, Model 1 as baseline 0); (5) Early Clinical Utility (Net Benefit at 10% threshold); and (6) Overall Discrimination (Integrated Discrimination Improvement, IDI). The expanded red polygon indicates the superior, multidimensional performance of Model 3. AUC, area under the receiver operating characteristic curve; NRI, Net Reclassification Improvement; IDI, Integrated Discrimination Improvement.

**Table 3:** Comprehensive performance comparison of three incremental models.

Metric	Model 1 (95% CI)	Model 2 (95% CI)	Model 3 (95% CI)	*P* Value
**C-index** ^1^	0.625 (0.567, 0.684)	0.673 (0.620, 0.725)	0.688 (0.636, 0.739)	0.1176
**AUC** ^1^	0.646 (0.576, 0.716)	0.694 (0.633, 0.755)	0.713 (0.651, 0.774)	<0.001
**NRI** ^2^	Reference	0.214 (0.070,0.334)	0.274 (0.137, 0.400)	<0.001
**IDI** ^2^	Reference	0.027 (0.009, 0.066)	0.037 (0.019, 0.088)	<0.001
**Brier Score** ^3^	0.108 (0.088, 0.127)	0.108 (0.088, 0.127)	0.108 (0.088, 0.127)	0.238
**Net Benefit** ^4^	0.032	0.045	0.047	—

1 C-index and 6-Month time-dependent AUC evaluate the overall and peak-time discriminative capabilities of the models;.

2 Continuous NRI and IDI quantify the incremental reclassification accuracy. Values for Model 2 and Model 3 represent absolute improvements calculated with Model 1 serving as the baseline reference. P-values indicate statistical significance for these improvements;.

3 Brier Score measures overall model calibration and accuracy (a lower score indicates superior calibration);.

4 Net Benefit (NB) derived from DCA reflects the absolute clinical utility at a representative early-intervention threshold of 10%. In accordance with established DCA methodological guidelines, statistical P-values are not computed for isolated threshold points; instead, clinical utility is assessed continuously across a range of threshold probabilities;.

In terms of overall discrimination, the baseline clinical model (Model 1) was upgraded to Model 2, and the discriminative performance of the model was moderately improved (C-index increased from 0.625 to 0.673; 6-month time-dependent AUC increased from 0.646 to 0.694). Notably, when systemic inflammatory biomarkers and acute radiation esophagitis were integrated into the ultimate comprehensive model (Model 3), the predictive efficiency improved. The C-index reached 0.688, and the 6-month AUC reached 0.713, compared with the baseline model. The 6-month time-dependent AUC represented a significant improvement over the baseline model (P < 0.05), confirming the independent prognostic value of the inflammatory markers.

To further evaluate the clinical error-correction ability of the new indicators, we calculated the Net Reclassification Improvement (NRI) and the Integrated Discrimination Improvement (IDI). Compared to the baseline reference (Model 1), Model 2 provided a 21.4% reclassification improvement. The fully-integrated Model 3 demonstrated discrimination, achieving a continuous NRI of 27.4% (P < 0.001) and an IDI increase of 3.7% (P < 0.001). These reclassification indices show that integrating systemic inflammatory indicators into traditional clinical evaluations may effectively reduce misclassification.

### 3.4 Calibration and decision-making curve analysis

The Brier score of Model 3 was 0.108, compared with 0.112 for the null model, indicating a small reduction in overall prediction error. Bootstrap-corrected calibration curves showed generally acceptable agreement between predicted and observed risks (**Supplementary Table S4**). To assess model calibration, a calibration curve was constructed for the Model 3 at 6 months (**Supplementary Figure S3**). The bootstrap-corrected calibration slopes for each model are presented in **Supplementary Table S5**. Quintile-level calibration analysis in **Supplementary Table S6**.

In addition, this study uses decision curve analysis (DCA) to translate the above statistical accuracy into real-world clinical utility **Figure 7**. At the representative 10% early clinical intervention threshold, Model 3 provides the highest absolute net clinical benefit (0.047), outperforming Model 2 (0.045) and Model 1 (0.032). These findings suggest that Model 3 may provide greater net benefit than the simpler models within the evaluated threshold probability range.

**Figure 7:**
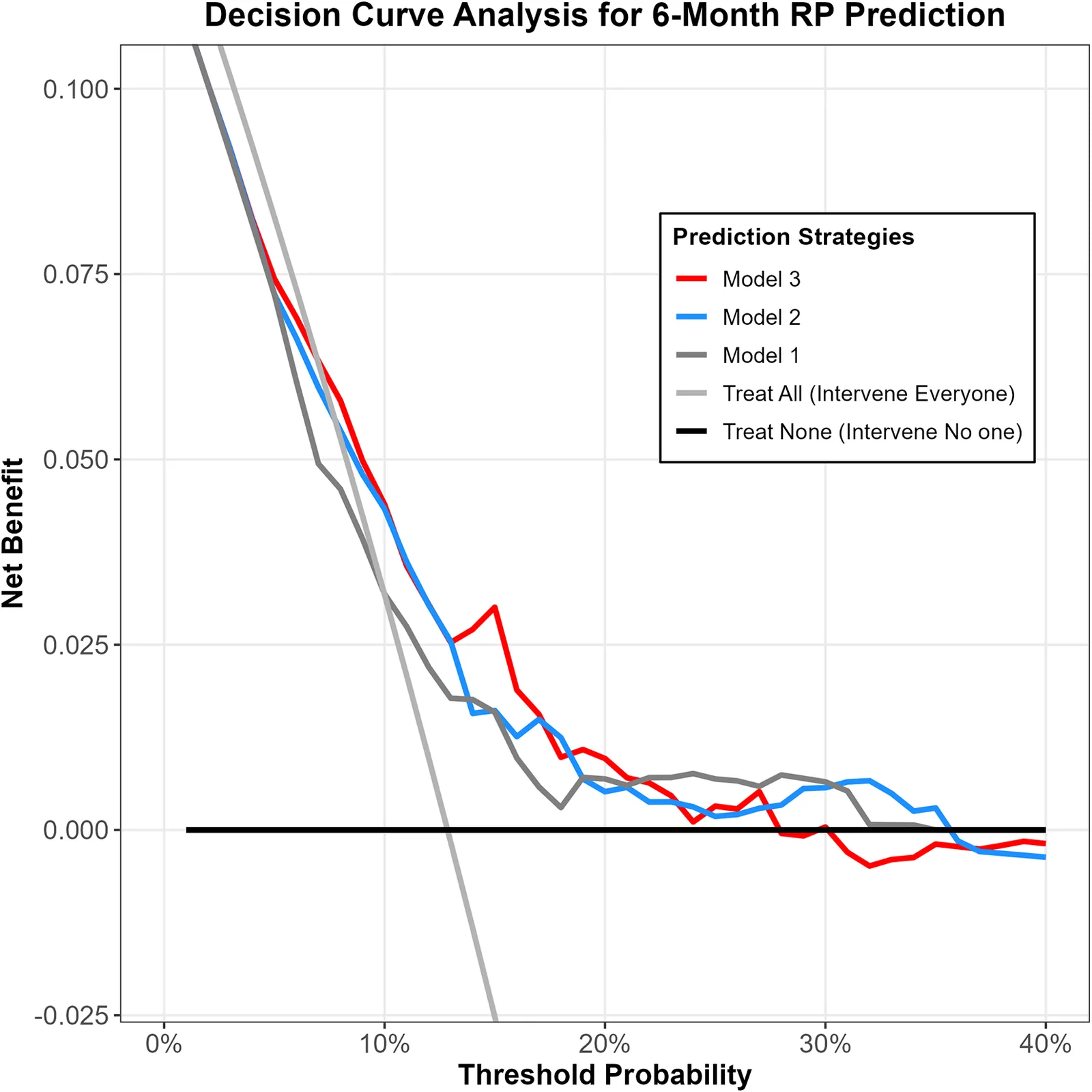
Decision curve analysis (DCA) of the three sequential models. The net benefit (y-axis) is plotted against the threshold probability (x-axis). The performance of Model 3 (red line) is compared with Model 2 (blue line), Model 1 (dark gray line), and the default strategies of intervening for all patients (light gray line) or no patients (black horizontal line).

### 3.5 SHAP analysis

To improve model interpretability, SHAP analysis was performed directly on the final Cox proportional hazards model (**Figure 8**). SHAP values were calculated on the model linear indicator (log-hazard) scale, and beeswarm and waterfall plots were generated to visualize global and individual factor contributions, respectively.

**Figure 8:**
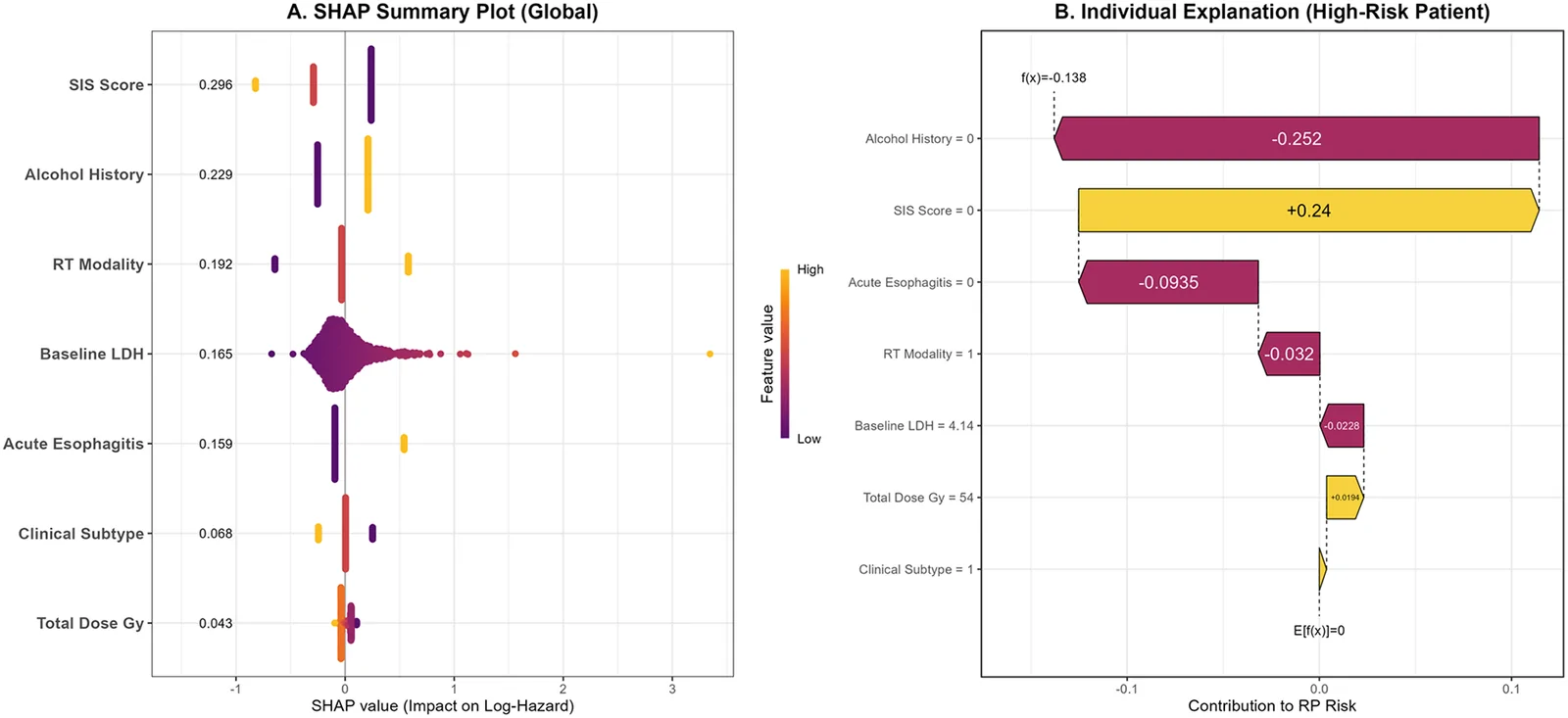
SHAP-based global and local interpretability analysis of the comprehensive model. **(A)** SHAP summary beeswarm plot demonstrating global feature importance. Features on the y-axis are ranked from top to bottom by their mean absolute SHAP values (displayed on the left). Each dot represents an individual patient. The x-axis indicates the SHAP value, where positive values drive a higher predicted risk of severe RP. The color gradient from purple to yellow/red represents the original feature value from low to high. **(B)** SHAP waterfall plot for a representative high-risk patient, selected for having a verified severe RP outcome and a predicted risk score within the top quartile (>75th percentile) of the cohort. The plot illustrates how individual features sequentially push the prediction from the cohort’s baseline expected value *E*[*f*(*x*)]=0 to the patient’s final individualized risk score f(x)= -0.138. Yellow arrows pushing rightward indicate risk-increasing contributions, while purple arrows pulling leftward indicate risk-decreasing contributions.

The global SHAP summary plot (**Figure 8A**) ranked the predictive importance of the variables, with the Systemic Inflammation Score (SIS Score) emerging as the primary driver (mean absolute SHAP: 0.296), followed by alcohol history (0.229), radiotherapy modality (0.192), baseline LDH (0.165), acute esophagitis (0.159), clinical subtype (0.068), and total radiation dose (0.043). Directionally, low values of the SIS Score (SIS = 0) and the presence of alcohol history, advanced radiotherapy modalities, elevated baseline LDH, and acute esophagitis were associated with increased RP risk. At the individual level, a SHAP waterfall plot of a representative patient (**Figure 8B**) illustrated personalized clinical decision-making, where the baseline expected log-hazard of 0 was adjusted by specific feature weights to predict a final log-hazard of -0.138. For this patient, the absence of alcohol history and acute esophagitis served as key protective factors, whereas an optimal baseline immune status (SIS Score = 0) and the radiotherapy dose (54Gy) were the main risk-promoting drivers.

## 4 Discussion

In this study, we developed and validated a combined model for early screening and risk assessment of radiation pneumonitis (RP) in lung cancer patients. By integrating systemic immune nutrition biomarkers LDH and SIS scores at baseline with early acute toxicity (radiation esophagitis), the comprehensive model achieved moderate discriminative performance (C-index: 0.688, 95% CI: 0.636, 0.739; 6-month AUC: 0.713, 95% CI: 0.651, 0.774). Our model’s overall discriminative performance appears comparable to that of several existing models in the literature (26–29). Our stepwise incremental value analysis indicated that the inclusion of systemic inflammatory biomarkers enhanced the model’s risk identification and assessment efficiency. This finding was further supported by SHAP analysis. These results suggest that incorporating systemic inflammatory biomarkers into existing baseline risk frameworks may improve identification accuracy, supporting the value of biologically integrated approaches in RP risk assessment (30).

At the same time, our SHAP interpretability analysis indicated that a Systemic Inflammation Score of zero (SIS = 0), representing a relatively favorable baseline inflammatory and nutritional status, was associated with a higher risk contribution to radiation pneumonitis. Some studies have suggested that acute radiation-induced lung injury may involve acute cytokine-mediated inflammation and T-cell-mediated immune responses (31–34). While it is theoretically possible that patients with preserved baseline immunity possess a greater capacity to mount robust, cytokine-mediated inflammatory responses following radiation-induced tissue injury. At the same time, patients in optimal baseline health might have been selected for more aggressive treatment protocols, such as concurrent chemoradiotherapy or intensive consolidation regimens, which could independently elevate their risk of pulmonary toxicity. Given the retrospective design of our study, this association should be regarded as a hypothesis-generating finding that requires independent validation in future prospective cohorts with detailed biological evaluations. In addition, elevated baseline LDH levels may reflect pre-existing tissue hypoxia and systemic tumor burden (35–37), which collectively promote fibrosis and inflammatory reactions within the pulmonary microenvironment.

The development of acute radiation esophagitis (RE) during treatment was found to be associated with a higher subsequent risk of RP, suggesting that on-treatment toxicities could serve as early indicators for risk stratification (38, 39). Given the time-dependent nature of this variable, a predefined 30-day landmark design was implemented before model development, ensuring that all candidate factors were ascertained prior to the subsequent occurrence of RP. Therefore, the proposed nomogram should be regarded as a dynamic monitoring tool for use during the early course of radiotherapy. In addition to the anatomical overlap of radiation fields, the rapid emergence of mucosal toxicity is an important warning signal that reflects individual susceptibility to treatment-related toxicity (22). The emergence of acute radiation esophagitis during radiotherapy may serve as a surrogate marker of individual radiosensitivity or reflect anatomical proximity of the esophagus to high-dose radiation fields, warranting further investigation. The incorporation of this early clinical indication into our nomogram has the potential to facilitate risk identification prior to the irreversible onset of severe pulmonary fibrosis.

Additionally, in our multivariable analysis, we identified alcohol history and radiotherapy modality as two important independent indicators for RP risk identification. One of the key findings is that a history of alcohol consumption was associated with an increased risk of RP. The underlying mechanism for this association is likely multifactorial. Chronic alcohol use may increase susceptibility to radiation-induced lung injury by inducing systemic inflammation, impairing immune function, and disrupting alveolar macrophage functions (40, 41). This finding suggests that alcohol history should be considered as a relevant factor in routine risk assessment for RP.

Another finding suggests a possible association between the type of radiation therapy and the risk of RP, specifically the higher risk observed with Tomo therapy compared to IMRT. The higher risk of radiation pneumonitis with Tomo Therapy may be attributed to its 360-degree helical delivery method, which typically increases the low-dose lung volume (V5) despite sparing high-dose regions, increasing the risk for triggering radiation pneumonitis (4, 42, 43). However, this proposed mechanism is based on retrospective data and requires validation in future prospective trials with detailed dosimetric analysis.

Our variable selection process utilized a hybrid, semi-automated strategy that combined automated LASSO regression with manual clinical selection. While this semi-automated approach effectively resolves multicollinearity and clinical redundancy to improve the biological plausibility of our model, it may also introduce potential clinician bias during the manual filtering phase. Furthermore, the addition of the newly identified biomarkers to the baseline clinical model yielded a continuous NRI of 0.274 (27.4%), supporting improved risk reclassification for RP compared with the baseline model alone (44). Decision Curve Analysis (DCA) further confirms the clinical applicability of the model. The model provided the greatest net benefit within the clinically relevant intervention threshold range of 10 percent to 15 percent, suggesting that it may assist in identifying patients who could benefit from intensified monitoring. However, because DCA was performed at a single prespecified time point, the estimated net benefit reflects clinical utility only at that stage. Whether the model provides similar clinical benefit throughout the course of radiotherapy and follow-up warrants further investigation using dynamic monitoring approaches.

There are several limitations in this study that warrant discussion. First, radiotherapy dosimetric parameters, including mean lung dose, V20, and V5, were unavailable in this retrospective dataset due to historical database constraints. Consequently, we chose to exclude these incomplete variables to maintain a robust study population, meaning our proposed nomogram should be viewed as an auxiliary clinical biological tool for early risk screening and identification. Second, heterogeneity was inevitable within this real-world cohort, primarily encompassing variations in patient selection, radiotherapy modalities, and systemic treatment strategies. Consequently, our findings should be considered preliminary, and future prospective, multicenter studies with standardized protocols are highly warranted to validate and refine this risk assessment system in more homogeneous patient populations.

## 5 Conclusion

In conclusion, we developed an explainable nomogram incorporating clinical characteristics, systemic inflammatory indicators, and early radiation esophagitis for risk assessment of radiation pneumonitis in patients with lung cancer. The model demonstrated modest discriminatory ability and generally acceptable calibration. Competing-risk analysis supported the consistency of the identified associations, and SHAP analysis enhanced the interpretability of the model. Given its reliance on routinely available clinical variables, the nomogram may serve as an auxiliary tool for early RP risk assessment. Further prospective, multicenter validation incorporating standard dosimetric parameters is warranted to establish its generalizability and clinical utility.

## Data Availability

The raw data supporting the conclusions of this article will be made available by the authors, without undue reservation.

## Footnotes

1 LDH levels were rescaled by dividing by 50 for ease of interpretation of the hazard ratio.

2 SIS was analysed as a predefined categorical variable (scores 0–2); therefore, no data-driven threshold selection was performed.

## Glossary

RP: radiation pneumonitis
SHAP: SHapley Additive exPlanations
AUC: the area under the receiver operating curve
NRI: Net Reclassification Improvement
IDI: Integrated Discrimination Improvement
DCA: decision curve analysis
LDH: lactate dehydrogenase
SIS: Systemic Inflammation Score
RE: radiation esophagitis
RT: Radiotherapy
NSCLC: non-small cell lung cancer
SCLC: small cell lung cancer
CCRT: chemoradiotherapy
NLR: neutrophil-to-lymphocyte ratio
dNLR: derived NLR
PLR: platelet-to-lymphocyte ratio
SII: systemic immune inflammatory index
SIRI: systemic inflammatory response index
ECOG PS: Eastern Cooperative Oncology Group performance status
CTCAE: Common Terminology Criteria for Adverse Events
NCI: National Cancer Institute
MICE: multiple imputation by chained equations
IQR: interquartile range
ROC: receiver operating characteristic
CIF: cumulative incidence functions
ALB: albumin
V20: the percentage of the total lung volume receiving 20 Gy or more
IMRT: intensity-modulated radiotherapy
VMAT: volumetric modulated arc therapy
TOMO: helical tomotherapy
BMI: body mass index
HR: hazard ratio
sHR: subdistribution hazard ratio
CI: confidence intervals.
