# Supplementary material for: An explainable nomogram incorporating systemic inflammatory indicators and radiation esophagitis for risk assessment of radiation pneumonitis in lung cancer
Source: Front Immunol. 2026 Sep 25;17:1910553. doi: 10.3389/fimmu.2026.1910553 (PMC13650004; doi:10.3389/fimmu.2026.1910553)
Supplement: Supplementary file 1 [file DataSheet1.pdf]

Supplementary Material

1 Supplementary Figures and Tables

1.1 Supplementary Tables

Supplementary Table S1. Distribution of Patients by Risk Groups Based on Optimal Cutoffs.

Characteristic	Overall (N = 726)	With RP (N = 86)	Without RP （N = 640）	*P* value
**ALB**				<0.001
<41.35	290 (39.9%)	20 (23.3%)	270 (42.2%)	
≥41.35	436 (60.1%)	66 (76.7%)	370 (57.8%)	
**PNI**				0.003
<48.52	274 (37.7%)	20 (23.3%)	254 (39.7%)	
≥48.52	452 (62.3%)	66 (76.7%)	386 (60.3%)	
**LDH**				0.015
<204.95	388 (53.4%)	35 (40.7%)	353 (55.2%)	
≥204.95	338 (46.6%)	51 (59.3%)	287 (44.8%)	
**Age**				0.015
<64.5	323 (44.5%)	49 (57.0%)	274 (42.8%)	
≥64.5	403 (55.5%)	37 (43.0%)	366 (57.2%)	
**Hb**				0.013
<128.45	453 (62.4%)	43 (50.0%)	410 (64.1%)	
≥128.45	273 (37.6%)	43 (50.0%)	230 (35.9%)	
**Total Dose(Gy)**				0.014
<54.5	282 (38.8%)	44 (51.2%)	238 (37.2%)	
≥54.5	444 (61.2%)	42 (48.8%)	402 (62.8%)	
**ALI**				0.031
<18.84	84 (11.6%)	4 (4.7%)	80 (12.5%)	

Supplementary Table S1 Continued

Characteristic	Overall<br>(N = 726)	With RP<br>(N = 86)	Without RP<br>（N = 640）	*P* value
≥18.84	642 (88.4%)	82 (95.3%)	560 (87.5%)	
**CRE**				0.029
<70.05	375 (51.7%)	54 (62.8%)	321 (50.2%)	
≥70.05	351 (48.3%)	32 (37.2%)	319 (49.8%)	
**ANC**				0.081
<3.55	414 (57.0%)	57 (66.3%)	357 (55.8%)	
≥3.55	312 (43.0%)	29 (33.7%)	283 (44.2%)	
**dNLR**				0.174
<1.1	230 (31.7%)	33 (38.4%)	197 (30.8%)	
≥1.1	496 (68.3%)	53 (61.6%)	443 (69.2%)	
**NLR**				0.034
<5.39	652 (89.8%)	83 (96.5%)	569 (88.9%)	
≥5.39	74 (10.2%)	3 (3.5%)	71 (11.1%)	
**ALC**				0.296
<1.19	189 (26.0%)	18 (20.9%)	171 (26.7%)	
≥1.19	537 (74.0%)	68 (79.1%)	469 (73.3%)	
**GLU**				0.084
<5.4	324 (44.6%)	46 (53.5%)	278 (43.4%)	
≥5.4	402 (55.4%)	40 (46.5%)	362 (56.6%)	
**PIV**				0.075
<725.66	639 (88.0%)	81 (94.2%)	558 (87.2%)	
≥725.66	87 (12.0%)	5 (5.8%)	82 (12.8%)	
**PLT**				0.413
<191.5	296 (40.8%)	39 (45.3%)	257 (40.2%)	
≥191.5	430 (59.2%)	47 (54.7%)	383 (59.8%)	

Supplementary Table S1 Continued

Characteristic	Overall (N = 726)	With RP (N = 86)	Without RP （N = 640）	*P* value
**MONO**				0.327
<0.3	105 (14.5%)	9 (10.5%)	96 (15.0%)	
≥0.3	621 (85.5%)	77 (89.5%)	544 (85.0%)	
**EOS**				0.353
<0.04	303 (41.7%)	40 (46.5%)	263 (41.1%)	
≥0.04	423 (58.3%)	46 (53.5%)	377 (58.9%)	
**PLR**				0.248
<142.31	402 (55.4%)	53 (61.6%)	349 (54.5%)	
≥142.31	324 (44.6%)	33 (38.4%)	291 (45.5%)	
**SII**				0.243
<270.6	192 (26.4%)	18 (20.9%)	174 (27.2%)	
≥270.6	534 (73.6%)	68 (79.1%)	466 (72.8%)	
**INR**				0.566
<0.96	400 (55.1%)	50 (58.1%)	350 (54.7%)	
≥0.96	326 (44.9%)	36 (41.9%)	290 (45.3%)	
**BMI**				0.320
<27.05	578 (79.6%)	65 (75.6%)	513 (80.2%)	
≥27.05	148 (20.4%)	21 (24.4%)	127 (19.8%)	
**BASO**				0.672
<0.01	152 (20.9%)	16 (18.6%)	136 (21.3%)	
≥0.01	574 (79.1%)	70 (81.4%)	504 (78.8%)	
**MLR**				0.086
<0.17	72 (9.9%)	4 (4.7%)	68 (10.6%)	
≥0.17	654 (90.1%)	82 (95.3%)	572 (89.4%)	
**SIRI**				0.248
<1.12	414 (57.0%)	44 (51.2%)	370 (57.8%)	

Supplementary Table S1 Continued

Characteristic	Overall (N = 726)	With RP (N = 86)	Without RP （N = 640）	*P* value
≥1.12	312 (43.0%)	42 (48.8%)	270 (42.2%)	
**dMLR**				0.909
<0.1	372 (51.2%)	45 (52.3%)	327 (51.1%)	
≥0.1	354 (48.8%)	41 (47.7%)	313 (48.9%)	
**PAR**				0.646
<5.01	375 (51.7%)	42 (48.8%)	333 (52.0%)	
≥5.01	351 (48.3%)	44 (51.2%)	307 (48.0%)	
**WBC**				0.260
<3.45	108 (14.9%)	9 (10.5%)	99 (15.5%)	
≥3.45	618 (85.1%)	77 (89.5%)	541 (84.5%)	
**LIPI**				0.450
Good	542 (74.7%)	62 (72.1%)	480 (75.0%)	
Intermediate	153 (21.1%)	22 (25.6%)	131 (20.5%)	
Poor	31 (4.3%)	2 (2.3%)	29 (4.5%)	
**SIS_score**				0.019
0	449 (61.8%)	65 (75.6%)	384 (60.0%)	
1	226 (31.1%)	18 (20.9%)	208 (32.5%)	
2	51 (7.0%)	3 (3.5%)	48 (7.5%)	

Continuous variables were compared based on cutoff values.

Supplementary Table S2. Univariate Cox Proportional Hazards Analyses of Baseline Characteristics and Inflammatory Biomarkers Associated with Symptomatic Radiation Pneumonitis.

**Variable**	**HR (95% CI)**	***P* value**
**Age (≥64.5)**	0.67 (0.44, 1.02)	0.063
**Gender**	1.02 (0.65, 1.60)	0.924
**BMI (≥27.05)**	1.28 (0.78, 2.10)	0.332
**Alcohol_History**	1.53 (0.98, 2.38)	0.057
**Diabetes**	0.87 (0.46, 1.65)	0.671
**Hypertension**	0.78 (0.43, 1.40)	0.391
**Obstructive Pneumonia**	0.93 (0.59, 1.48)	0.761
**Tumor Location**	0.96 (0.79, 1.17)	0.689
**Pathology Type**	0.83 (0.63, 1.09)	0.183
**Clinical Subtype**		0.255
I, II	Reference	
III	1.27 (0.63, 2.54)	
IV	0.71 (0.27, 1.92)	
**T Stage**		0.485
1	Reference	
2	1.66 (0.74, 3.72)	
3	1.28 (0.53, 3.10)	
4	1.23 (0.53, 2.83)	
**N Stage**		0.912

Supplementary Table S2 Continued

Variable	HR (95% CI)	*P* value
0	Reference	
1	1.14 (0.46, 2.80)	
2	1.00 (0.50, 1.99)	
3	1.19 (0.57, 2.49)	
**Metastatic**	0.58 (0.27, 1.25)	0.134
**RT Modality**	1.84 (1.24, 2.71)	0.008
VMAT	Reference	
IMRT	0.68 (0.31, 1.49)	
TOMO	2.01 (1.24, 3.25)	
**Total Dose (Gy) (≥54.5)**	0.64 (0.42, 0.98)	0.042
**Surgery_Flag**	1.35 (0.55, 3.33)	0.534
**Chemotherapy**	0.84 (0.53, 1.32)	0.454
**Immunotherapy**	1.19 (0.77, 1.84)	0.449
**Targeted Therapy**	0.81 (0.49, 1.34)	0.402
**Endocrine Therapy**	0.73 (0.47, 1.13)	0.165
**Esophagitis**	2.06 (1.26, 3.37)	0.007
**WBC (≥3.45)**	1.59 (0.80, 3.17)	0.161
**Hb (≥128.45)**	1.76 (1.15, 2.68)	0.009
**ALC (≥1.19)**	1.30 (0.77, 2.19)	0.309

Supplementary Table S2 Continued

Variable	HR (95% CI)	*P* value
**ANC (≥3.55)**	0.72 (0.46, 1.13)	0.146
**PLT (≥191.5)**	0.76 (0.50, 1.17)	0.216
**MONO (≥0.3)**	1.52 (0.76, 3.04)	0.206
**BASO (≥0.01)**	1.25 (0.72, 2.15)	0.414
**EOS (≥0.04)**	0.84 (0.55, 1.28)	0.416
**GLU (≥5.4)**	0.67 (0.44, 1.03)	0.066
**ALB (≥41.35)**	2.18 (1.32, 3.60)	0.001
**LDH (≥204.95)**	1.81 (1.18, 2.79)	0.006
**CRE (≥70.05)**	0.66 (0.42, 1.02)	0.057
**INR (≥0.96)**	0.90 (0.58, 1.38)	0.620
**NLR (≥5.39)**	0.33 (0.10, 1.05)	0.024
**dNLR (≥1.1)**	0.82 (0.53, 1.27)	0.389
**SIRI (≥1.12)**	1.35 (0.89, 2.07)	0.159
**MLR (≥0.17)**	2.39 (0.88, 6.52)	0.051
**dMLR (≥0.1)**	0.88 (0.57, 1.34)	0.542
**PLR (≥142.31)**	0.79 (0.51, 1.23)	0.295
**SII (≥270.6)**	1.46 (0.87, 2.46)	0.137
**PNI (≥48.52)**	1.93 (1.17, 3.19)	0.006
**PAR (≥5.01)**	1.27 (0.67, 2.39)	0.448

Supplementary Table S2 Continued

**Variable**	**HR (95% CI)**	***P* value**
**ALI (≥18.84)**	2.62 (0.96, 7.14)	0.029
**PIV (≥725.66)**	0.46 (0.19, 1.13)	0.056
**LIPI**		0.344
Good	Reference	
Intermediate	1.37 (0.84, 2.23)	
Poor	0.63 (0.16, 2.59)	
**SIS**		0.037
0	Reference	
1	0.55 (0.33, 0.93)	
2	0.50 (0.16, 1.58)	

Continuous variables were compared based on cutoff values.

Supplementary Table S3. Univariate Fine, Gray Competing Risks Analyses of Baseline Factors Associated with Symptomatic Radiation Pneumonitis.

Variable	sHR (95% CI)	*P* value
**Age**	0.67 (0.44, 1.02)	0.063
**BMI**	1.28 (0.78, 2.09)	0.326
**Gender**	1.02 (0.66, 1.60)	0.920
**Alcohol History**	1.53 (0.98, 2.38)	0.061
**Diabetes**	0.87 (0.47, 1.64)	0.678
**Hypertension**	0.78 (0.43, 1.40)	0.402
**Obstructive Pneumonia**	0.93 (0.59, 1.48)	0.768
**Surgery Flag**	1.35 (0.55, 3.28)	0.514
**Chemotherapy**	0.84 (0.53, 1.32)	0.445
**Immunotherapy**	1.19 (0.77, 1.84)	0.440
**Targeted Therapy**	0.81 (0.49, 1.34)	0.412
**Endocrine Therapy**	0.73 (0.47, 1.13)	0.158
**Esophagitis**	2.05 (1.27, 3.31)	0.004
**RT Modality**		
VMAT	Reference	
IMRT	0.58 (0.28, 1.21)	0.147
TOMO	2.12 (1.33, 3.38)	0.002
**Total Dose(Gy)**	0.64 (0.42, 0.98)	0.041
**Tumor Location**	0.96 (0.79-1.16)	0.676
**Pathology Type**	0.83 (0.63-1.08)	0.157
**Clinical Subtype**		
I, II	Reference	
III	1.50 (0.87, 2.57)	0.143
IV	0.58 (0.26, 1.27)	0.173
**T Stage**		

Supplementary Table S3 Continued

Variable	sHR (95% CI)	*P* value
1	Reference	
2	1.38 (0.90, 2.11)	0.139
3	0.93 (0.55, 1.57)	0.773
4	0.86 (0.54, 1.36)	0.512
**N Stage**		
0	Reference	
1	1.07 (0.53, 2.16)	0.843
2	0.89 (0.58, 1.35)	0.584
3	1.17 (0.73, 1.87)	0.517
**Metastatic**	0.58 (0.26, 1.27)	0.173
**ALC**	1.30 (0.77, 2.18)	0.321
**ANC**	0.72 (0.46, 1.12)	0.149
**PLT**	0.76 (0.50, 1.17)	0.211
**WBC**	1.59 (0.80, 3.14)	0.184
**Hb**	1.75 (1.15, 2.67)	0.009
**MONO**	1.52 (0.77, 2.99)	0.223
**BASO**	1.25 (0.73, 2.12)	0.418
**EOS**	0.84 (0.55, 1.28)	0.410
**GLU**	0.67 (0.44, 1.02)	0.065
**ALB**	2.18 (1.32, 3.58)	0.002
**LDH**	1.81 (1.18, 2.78)	0.006
**CRE**	0.66 (0.42, 1.02)	0.059
**INR**	0.90 (0.59, 1.38)	0.624
**NLR**	0.33 (0.10, 1.05)	0.060
**dNLR**	0.82 (0.53, 1.27)	0.381
**SIRI**	1.35 (0.89, 2.06)	0.162

Supplementary Table S3 Continued

Variable	sHR (95% CI)	*P* value
**MLR**	2.39 (0.89, 6.42)	0.085
**dMLR**	0.88 (0.57, 1.34)	0.539
**PLR**	0.79 (0.52, 1.23)	0.299
**SII**	1.46 (0.87, 2.45)	0.152
**PNI**	1.93 (1.17, 3.17)	0.009
**PAR**	1.09 (0.72, 1.67)	0.674
**ALI**	2.61 (0.95, 7.17)	0.062
**PIV**	0.46 (0.19, 1.12)	0.088
**LIPI**		
Good	Reference	
Intermediate	1.40 (0.87, 2.25)	0.167
Poor	0.59 (0.14, 2.50)	0.474
**SIS**		
Score 0	Reference	
Score 1	0.58 (0.34, 0.97)	0.037
Score 2	0.58 (0.18, 1.87)	0.364

Continuous variables were compared based on cutoff values.

Supplementary Table S4. Comparison of Bootstrap, corrected Brier scores with the null model.

Model	Brier Score	Null Brier Score	Absolute Reduction	Scaled Brier Score
Model 1	0.1079	0.1120	0.0042	0.0374
Model 2	0.1079	0.1120	0.0044	0.0392
Model 3	0.1076	0.1120	0.0044	0.0395

Supplementary Table S5. Bootstrap, corrected calibration metrics for the three incremental predictive models.

Model	Calibration Slope
Model 1	0.8026
Model 2	0.8133
Model 3	0.8143

Supplementary Table S6. Quintile, based calibration of predicted versus observed 6, month radiation pneumonitis risk across three incremental predictive models.

Risk Quintile	n	Predicted Risk	Observed Risk	Absolute Difference
**Model 1**				
Q1 (lowest)	172	-0.0134	0.0165	0.0299
Q2	158	-0.0100	0.0211	0.0311
Q3	127	-0.0088	0.0113	0.0202
Q4	126	-0.0077	0.0136	0.0213
Q5 (highest)	143	-0.0073	0.0074	0.0147
**Model 2**				
Q1 (lowest)	146	-0.0323	-0.0080	0.0243
Q2	145	-0.0297	0.0016	0.0313
Q3	145	-0.0281	0.0157	0.0437
Q4	145	-0.0268	0.0307	0.0574
Q5 (highest)	145	-0.0248	0.0590	0.0838
**Model 3**				
Q1 (lowest)	146	-0.0481	0.0000	0.0481
Q2	145	-0.0448	0.0065	0.0514
Q3	145	-0.0421	0.0098	0.0518
Q4	145	-0.0369	0.0386	0.0755
Q5 (highest)	145	-0.0289	0.0668	0.0957

1.2 Supplementary Figures

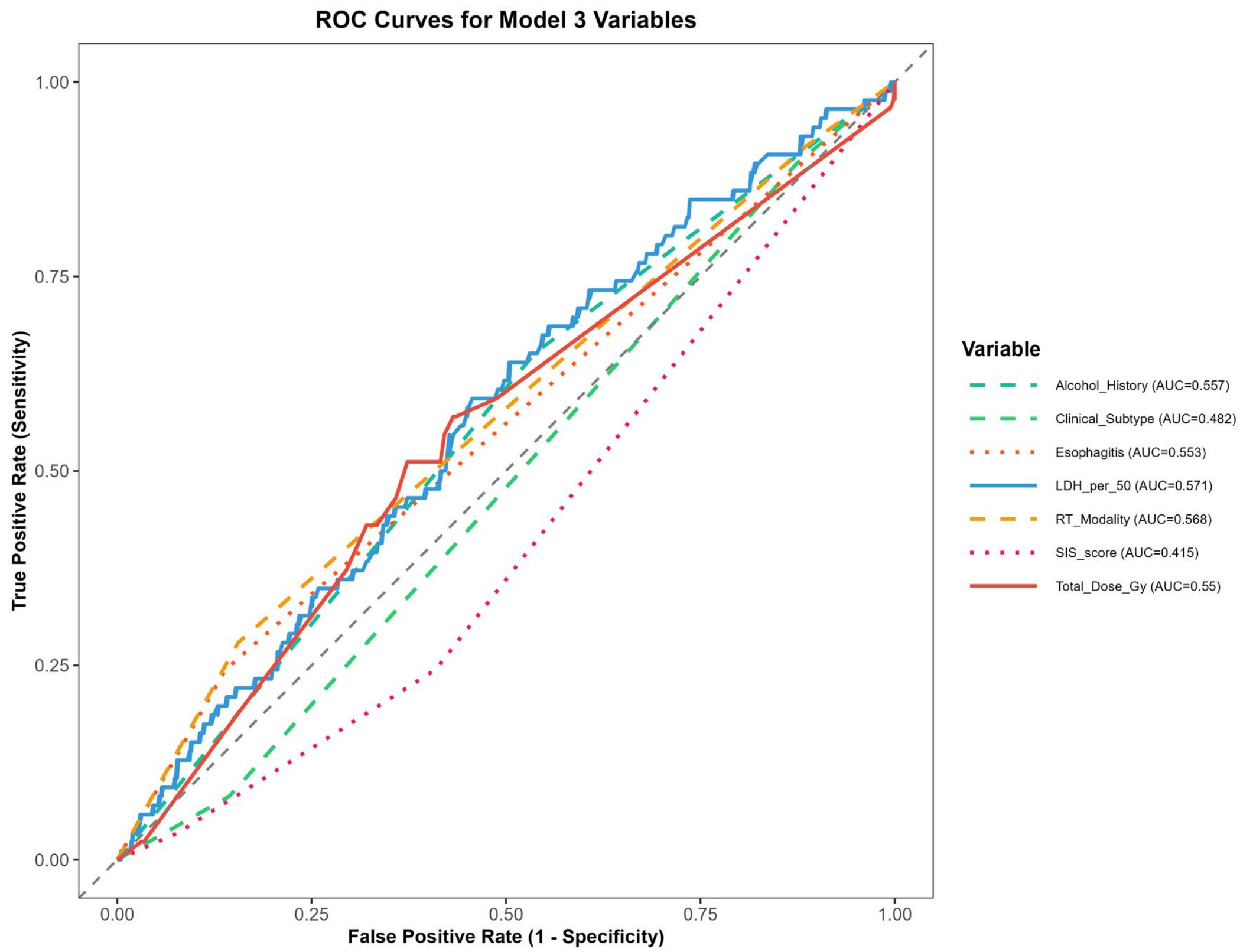

Supplementary Figure S1. Receiver operating characteristic (ROC) curves of candidate variables.

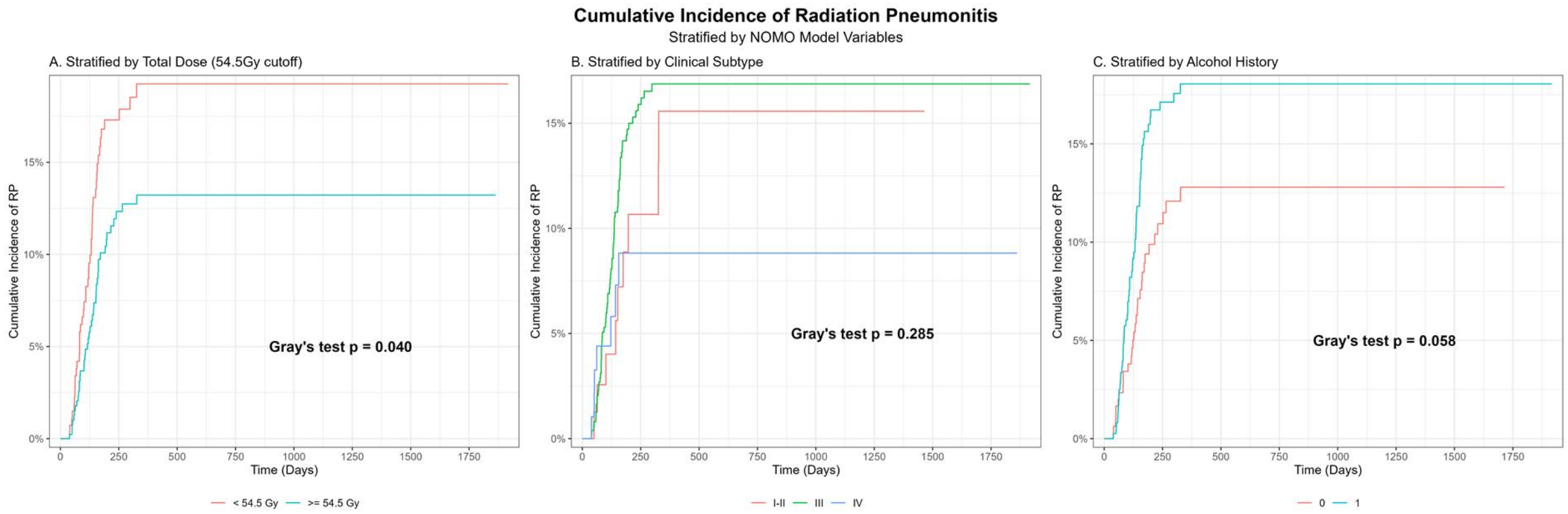

Supplementary Figure S2. Cumulative incidence of radiation pneumonitis (RP) according to baseline characteristics. Curves are stratified by (A) total radiation dose (cutoff at 54.5 Gy), (B) clinical subtype, (C) alcohol history. Gray’s test was used to assess differences between curves.

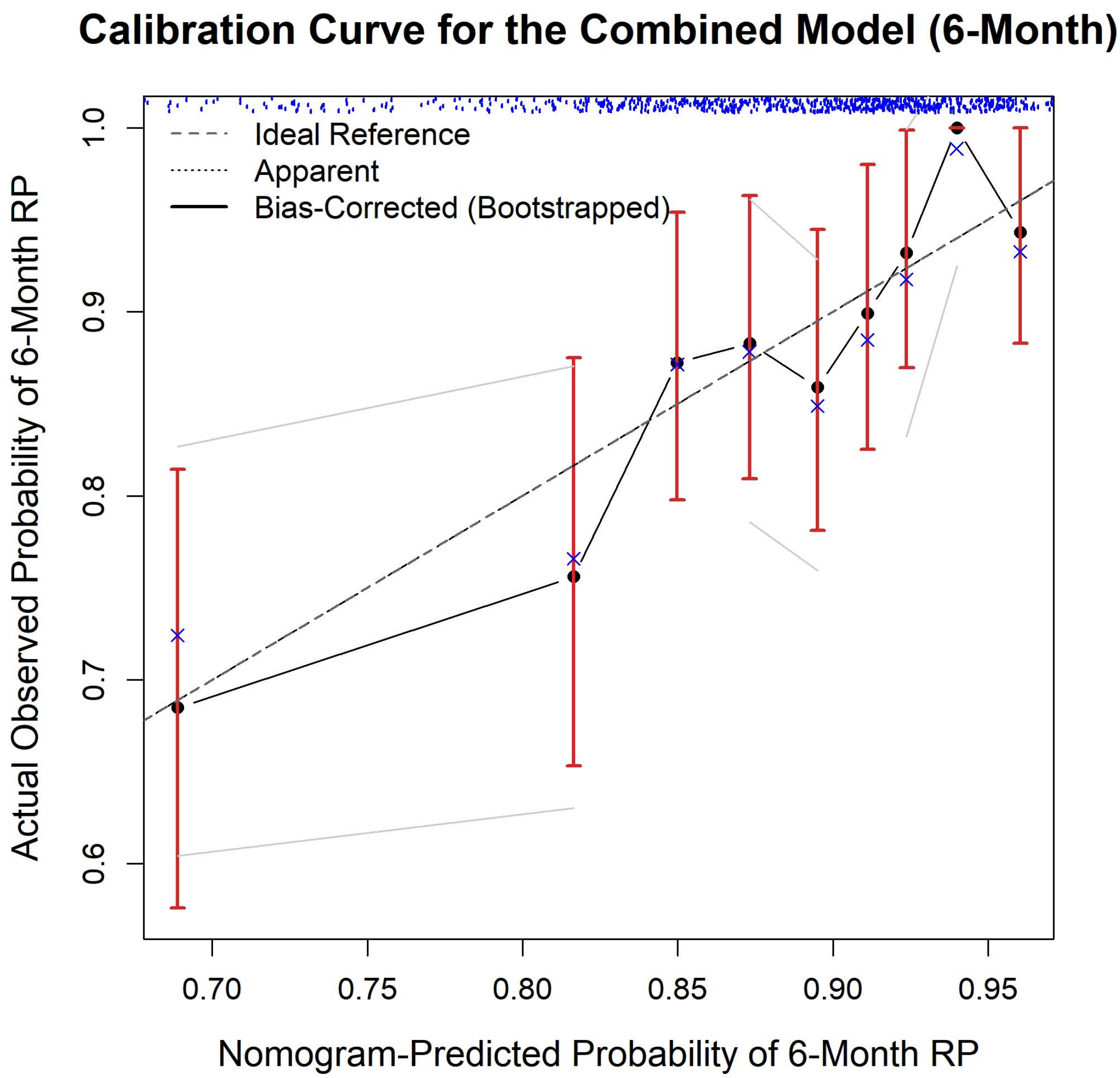

Supplementary Figure S3. Calibration curve of the Model 3 for predicting six month radiation pneumonitis free survival probability. The x axis represents the nomogram predicted probability of six month survival without radiation pneumonitis, and the y axis represents the actual observed survival probability. The diagonal grey dashed line represents the ideal reference where predicted and observed survival probabilities are identical. The dotted line represents the apparent model accuracy, and the solid black line represents the bias corrected calibration estimated through 1000 bootstrap resamples. The red vertical bars indicate the 95 percent confidence intervals of the observed survival estimates, and the blue rug plot at the top displays the density distribution of the patients in the cohort.
